# Supplementary material for: EnzML: multi-label prediction of enzyme classes using InterPro signatures
Source: BMC Bioinformatics. 2012 Apr 25;13:61. doi: 10.1186/1471-2105-13-61 (PMC3483700; doi:10.1186/1471-2105-13-61)
Supplement: Addtional file 5 — The Java code to format the data files, evaluate and predict. The file enzml_java_code.tar.gz contains the Java code used to format database data to ARFF and XML formats, to execute cross and train-test (jackknife) evaluations and to record evaluation results to database. More information is included in the readme.txt file and the Javadoc files. The code can be used with a MySQL database. To use a different database software, other JDBC drivers might be required. [file 1471-2105-13-61-S5.gz › java_code/enzml2011/doc/uk/ac/ed/inf/taweka/mulan/package-tree.html]

uk.ac.ed.inf.taweka.mulan Class Hierarchy


---


|  |  |  |
| --- | --- | --- |
| **Overview** \| **Package** \| Class \| Use \| **Tree** \| **Deprecated** \| **Index** \| **Help** | |  |
| **PREV**   **NEXT** | **FRAMES**    **NO FRAMES**     **All Classes** |  |


---


## Hierarchy For Package uk.ac.ed.inf.taweka.mulan

**Package Hierarchies:**: All Packages

---

## Class Hierarchy

- java.lang.Object
  - uk.ac.ed.inf.taweka.weka.**AttributeFactory**
    - uk.ac.ed.inf.taweka.mulan.**MulanAttributeFactory**
    - uk.ac.ed.inf.taweka.mulan.**AttributeUtils**- uk.ac.ed.inf.taweka.weka.**DataSetDbLoader**
        - uk.ac.ed.inf.taweka.mulan.**MulanDataSetDbLoader**
        - uk.ac.ed.inf.taweka.weka.**DataSetGenerator**
          - uk.ac.ed.inf.taweka.mulan.**MulanDataSetGenerator**
          - uk.ac.ed.inf.taweka.**Initialised** (implements uk.ac.ed.inf.taweka.Initialisable)
            - uk.ac.ed.inf.taweka.weka.**Arff**
              - uk.ac.ed.inf.taweka.mulan.**MulanArff**
              - uk.ac.ed.inf.taweka.weka.**ArffProperties**
                - uk.ac.ed.inf.taweka.mulan.**MulanArffProperties**
            - uk.ac.ed.inf.taweka.weka.**InstancesFiller**
              - uk.ac.ed.inf.taweka.mulan.**MulanInstancesFiller**
              - uk.ac.ed.inf.taweka.mulan.**InstanceUtils**- uk.ac.ed.inf.utils.database.Manager
                  - uk.ac.ed.inf.taweka.weka.**DataSetManager**
                    - uk.ac.ed.inf.taweka.mulan.**MulanDataSetManager**

---


|  |  |  |
| --- | --- | --- |
| **Overview** \| **Package** \| Class \| Use \| **Tree** \| **Deprecated** \| **Index** \| **Help** | |  |
| **PREV**   **NEXT** | **FRAMES**    **NO FRAMES**     **All Classes** |  |


---
